# Cutting Edge: Synapse Propensity of Human Memory CD8 T Cells Confers Competitive Advantage over Naive Counterparts

**DOI:** 10.4049/jimmunol.1801687

**Published:** 2019-06-14

**Authors:** Viveka Mayya, Edward Judokusumo, Enas Abu-Shah, Willie Neiswanger, Chirag Sachar, David Depoil, Lance C. Kam, Michael L. Dustin

**Affiliations:** * Kennedy Institute of Rheumatology, University of Oxford, OX3 7FY Oxford, United Kingdom;; † Skirball Institute of Biomolecular Medicine, New York University School of Medicine, New York, NY 10016;; ‡ Department of Biological Engineering, Columbia University, New York, NY 10027; and; § Machine Learning Department, Carnegie Mellon University, Pittsburgh, PA 15213

## Abstract

Memory T cells are endowed with multiple functional features that enable them to be more protective than naive T cells against infectious threats. It is not known if memory cells have a higher synapse propensity (SP; i.e., increased probability to form immature immunological synapses that then provide an entry into different modes of durable interaction with APCs). In this study, we show that only human memory CD8 T cells have remarkably high SP compared with naive counterparts. Such a dichotomy between naive and memory cells is not observed within the human CD4 or murine CD8 T cell population. Higher SP in human memory CD8 T cells allows them to outcompete and prevent naive CD8 T cells from getting recruited to the response. This observation has implications for original antigenic sin and aging of the immune system in humans.

## Key Points

- Microcontact printing can be used to mimic spatially limiting Ag presentation.
- High synapse propensity of human memory CD8 T cells prevents naive cell recruitment.

## Introduction

Memory T cells exhibit functional avidity maturation that enables them to produce more cytokines, and sometimes undergo more clonal expansion, than naive cells at lower doses of Ag (1, 2). Furthermore, they produce cytokines more quickly in response to Ag (3), have reduced requirements for costimulation (4), and show multifunctionality typically absent in freshly primed T cells (5). All these properties contribute to enhanced protective function of memory T cells along with their increased precursor frequency.

T cell–intrinsic mechanisms responsible for these enhanced functionalities of memory T cells have not been clearly established. Intriguingly, all of the recent studies point to diminished output of TCR signaling in memory cells (6–8). An immediate outcome of Ag recognition and suprathreshold TCR signaling in rapidly migrating, poorly adhesive T cells is the formation of an immature immunological synapse (IS) (9). The immature IS refers to a phase of interaction lasting a few minutes, characterized by rapid spreading and adhesion (10). This transient but committed phase further develops into a stable, mature IS lasting over an hour or a motile immunological kinapse (IK) that can nonetheless result in durable interactions with APCs through confined migration (10, 11). We define the capability to form an immature IS as synapse propensity (SP) and consider it as an intrinsic property of a T cell. SP determines the fraction of precursor cells participating in the response. Therefore, it is appealing to consider the possibility that memory T cells have enhanced SP compared with the naive cells. However, diminished output of TCR signaling in memory T cells may also result in lower SP. We measured SP of naive and memory cells from different T cell subsets by multiple approaches. Among the subsets that we have examined, only the human memory CD8 T (CD8^+^ hTm) cells exhibit appreciably higher SP than naive counterparts. We have explored the consequence of higher SP in an ex vivo setting that mimics spatially limiting Ag presentation. We find that higher SP of CD8^+^ hTm cells gives them a competitive advantage at the expense of naive T cells.

## Materials and Methods

### Isolation of resting T cell subsets

Nonclinical and deidentified leukapheresis products were used as a source of resting human T cells. This was exempt from Institutional Review Board review at the New York University Medical Center and was approved at the University of Oxford (National Health Service Research Ethics Committee 11/H0711/7). Resting human T cell subsets were isolated using EasySep (STEMCELL Technologies) negative selection kits as described (11). Typical purity of naive and memory subsets is >90% after isolation (Supplemental Fig. 1). The New York University Medical Center Institutional Animal Care and Use Committee approved (Protocol 150609-01) experiments involving mice. Naive (CD44-ve) and *Listeria*-specific (CD44+ve) memory CD8 T cells were isolated by flow-cytometric sorting (FACSAria; BD Biosciences) from spleens of ∼12-wk-old B6 male mice 30–40 d postinfection with 5000 CFU of *L. monocytogenes*.

### Quantification of SP

Uniformly coated stimulatory surfaces, microcontact printed arrays of stimulatory spots, and bilayers were prepared and used for live imaging (30× silicone oil, 1.05 numerical aperture; Olympus) as described (11). Differential interference contrast micrographs in time-lapse series were used for detecting and tracking cells. Interference reflection microscopy images were used for ascertaining spreading or attachment. Naive and memory subsets were imaged together after differentially labeling with CellTracker dyes (Life Technologies). Time-lapse sequences were preprocessed in ImageJ, and the cells were tracked using the Tool for Integrative Analysis of Motility (11, 12). Bespoke scripts were written in MATLAB for the calculation of various correlates of SP. The software code, criteria, and algorithmic steps for the calculation of these correlates are available on GitHub (https://github.com/uvmayya/synapsePropensity). Total internal reflection fluorescence (TIRF) images (150×, 1.45 numerical aperture; Olympus) of fixed cells on bilayers were analyzed in ImageJ.

### Naive T cell activation in a competitive setting

On average, 1.15 T cells arrest per 10-μm spot, and 4.5 T cells arrest per 20-μm spot (11). To create competition, we used ∼120,000 cells for 63,000 10-μm spots or 180,000 cells on 22,800 20-μm spots. This is referred to as “1×” number of cells in Fig. 3. After 10–12 h, the cells were collected using ice-cold PBS containing 0.5% BSA and 2 mM EDTA. Activation was assessed based on flow cytometry after staining with anti-CD45RO and CD62L Abs. Our isolation procedure for CD8^+^ hTm cells provides the relevant memory subsets for competition, as in the human lymph nodes (13) (Supplemental Fig. 1).

### Statistical analyses

Statistical significance of differences in values, where a pair of values represent the T cell subsets of a donor, was calculated by paired *t* test (Prism; Graphpad). The *p* values from two-tailed tests are denoted as follows in the figures: *p < 0.05, **p < 0.01, ***p < 0.001, and ****p < 0.0001. If the pairing itself was found to be significant (i.e., *p* < 0.05), the asterisk rating above the plot is given within parentheses.

## Results

### CD8^+^ hTm cells have very high SP

**FIGURE 1.**
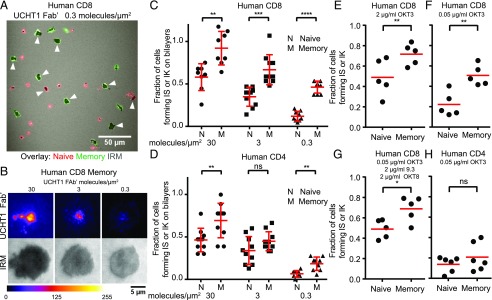
CD8^+^ hTm cells have high SP on uniform stimulatory surfaces. (**A**) CD8^+^ hTm (in green) and naive (in red) cells forming IS or IK (dark patches) on bilayers presenting 0.3 UCHT1 molecules/μm^2^. Attachment, along with additional criteria, is used to count cells with IS or IK. Such cells are highlighted with a white triangle in this example. (**B**) Centralized accumulation of Alexa 568-UCHT1 Fab′ at 30, 3, and 0.3 molecules/μm^2^ on bilayers in fixed CD8^+^ hTm cells visualized by TIRF. (**C** and **D**) Fractions of naive and memory cells in the human CD8 (C) and CD4 (D) subsets forming IS or IK on bilayers at indicated densities of UCHT1 Fab′, with expected number of cells per field (typically 30) as the denominator. Eight to ten fields per condition and subset were imaged and plotted. The data shown are representative of two independent experiments. (**E**–**H**) Fractions of human naive and memory T cells forming IS or IK on uniformly coated surface with immobilized CCL21, ICAM1, and OKT3. Note the differences in OKT3 concentration [(E) versus (F)] used for coating, presence of anti-CD28 and anti-CD8 Abs (G), and the data for the CD8 versus CD4 subsets (H). Time-lapse data (1.5–2 h) was used to determine the number of cells forming IS or IK based on scoring for attachment and deceleration. Each data point represents a separate donor and independent experiment. Mean value is shown in red. *p < 0.05, **p < 0.01, ***p < 0.001, ****p < 0.0001.

We first assessed SP of CD8^+^ hTm and naive cells on supported lypid bilayers presenting freely mobile ICAM1 and UCHT1 Fab′. The fraction of cells that have passed through the phase of immature IS represents SP in this context, as spatially uniform ligands do not typically result in a competitive setting. Because immature IS represents a transient phase committed to durable interaction, we relied on identifying cells that have formed IS or IK after a period of interaction with ligands on bilayers. For unbiased and statistically rigorous counting of cells with IS or IK, we used differentially labeled mixtures of naive and memory cells, fixed them after 30 min of interaction with ligands on bilayers, and imaged multiple larger fields afforded by lower magnification (Fig. 1A). We used attachment features (area, shape, etc.) recorded by interference reflection microscopy to decide whether a cell has formed an IS or IK, as visualizing formation of a central supramolecular activation cluster under these settings was not possible. More memory CD8 T cells had attachment footprints typical of cells with IS or IK at very low density of UCHT1 Fab′ (Fig. 1A). To ascertain that these cells were indeed forming bona fide IS or IK, we imaged the same preparations at high resolution in TIRF mode. Central supramolecular activation cluster formation, as assessed by centralized accumulation of UCHT1 Fab′, happened efficiently over a 100-fold range of ligand density (Fig. 1B). We confirmed that all CD8^+^ hTm cells with an attachment footprint typical of IS had indeed formed IS at 0.3 molecules/μm^2^ of UCHT1 Fab′ (Supplemental Fig. 2). As expected, we found that SP increases with increasing surface density of UCHT1 Fab′ for all subsets examined (Fig. 1C, 1D). Overall, the memory cells have higher SP than naive counterparts. The difference in SP between naive and memory cells is more striking in the CD8 subset, and CD8^+^ hTm cells have appreciably higher SP than the human memory CD4 T cells on bilayers presenting freely mobile ligands.

We next assessed SP of CD8^+^ hTm and naive cells on uniformly coated surfaces presenting immobilized CCL21, ICAM1, and OKT3. We noted that both central and effector memory CD8 T cells express sufficient CCR7 to show robust chemokinesis on immobilized CCL21 (Supplemental Fig. 3). Thus, we can rule out the possibility of lack of motility indirectly promoting SP in memory cells. We proceeded to score the fraction of cells forming IS or IK on uniformly coated surfaces by live imaging based both on attachment and deceleration (or arrest). As in the case of bilayers, SP was proportional to OKT3 density, but the difference between naive and memory CD8 T cells was more pronounced with less OKT3 (Fig. 1E, 1F, Supplemental Video 1). High SP of CD8^+^ hTm cells was maintained even with costimulation from immobilized anti-CD28 (clone 9.3) and anti-CD8 (clone OKT8) Abs (Fig. 1G). No significant difference in SP was observed between naive and memory human CD4 cells (Fig. 1H). Overall, we conclude that CD8^+^ hTm cells have high SP on uniformly presented stimulatory surfaces.

**FIGURE 2.**
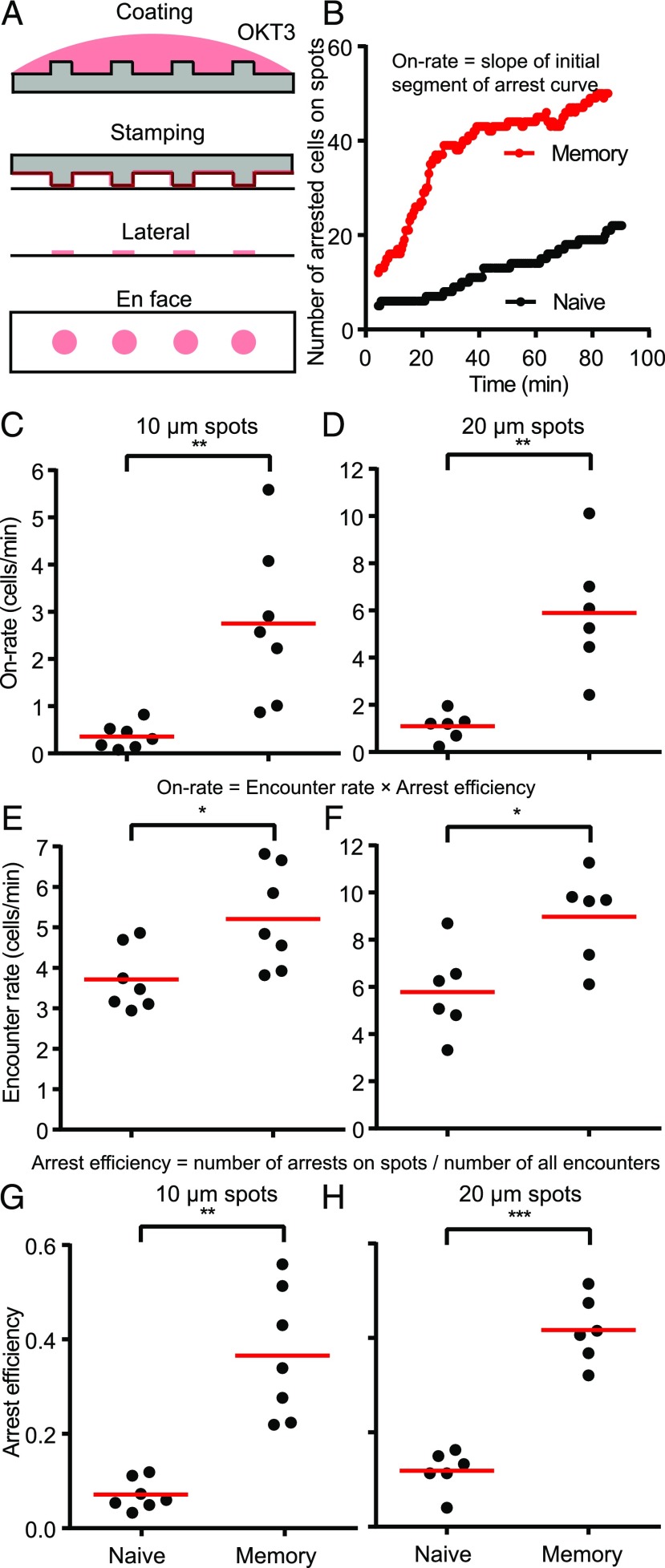
CD8^+^ hTm cells have high SP on stimulatory spots. (**A**) Schematic of the microcontact printing procedure. Ab is first adsorbed to polydimethylsiloxane (PDMS) casts that have short pillars with flat tops that will generate the spots. When the cast is laid on the coverglass, some of the Ab gets transferred from the PDMS surface onto the coverglass. However, this only happens at the top of the pillars. CCL21 and ICAM1 are then adsorbed uniformly across the surface from solution. (**B**) Number of CD8^+^ hTm and naive cells arresting on 10-μm spots that are 30 μm apart over 90 min. Sixty-four spots and typically at least the same number of cells were present in the field. (**C** and **D**) Rate of attachment (on-rate) of human naive and memory CD8 T cells on 10-μm spots that are 30 μm apart (C) and 20-μm spots that are 50 μm apart (D). The on-rate represents the values for the imaging field (50,625 μm^2^). (**E** and **F**) Rate of encounter of human naive and memory CD8 T cells with 10-μm (E) and 20-μm (F) spots. Again, the values given are for the imaging field, as above. (**G** and **H**) Arrest efficiency of human naive and memory CD8 T cells on 10-μm (G) or 20-μm (H) spots. Both encounter rate and arrest efficiency contribute to very high on-rate of attachment of CD8^+^ hTm cells compared with the naive cells. Each data point represents a separate donor and independent experiment in each plot. Mean value is shown in red. *p < 0.05, **p < 0.01, ***p < 0.001.

In vivo, both under the settings of priming in secondary lymphoid organs and during the early phase of recall response in peripheral tissues, Ag can be sparse and presented on few dispersed APCs in a spatially limiting manner (14). We have recapitulated this scenario ex vivo using the microcontact printing technology by creating stimulatory spots of OKT3 with pervasive ICAM1 and CCL21 (Fig. 2A) (11). CD8^+^ hTm cells rapidly attached and arrested on the stimulatory spots when compared with the naive CD8 T cells (Fig. 2B, Supplemental Video 2). The rate of arrest is the best measure of SP on spatially limiting stimulatory spots, as this represents a competitive setting. For an accurate measure of on-rate of arrest on the spots, we took the slope of the initial part of the attachment curve. We found that memory cells were capable of arresting on 10-μm spots at 7.1-fold and on 20-μm spots at 5.4-fold faster rates than naive cells (Fig. 2C, 2D). Treating the cell interactions with spots like a bimolecular reaction, the on-rate of arrest is the product of the rate of encounter with spots and arrest efficiency. The encounter rate quantifies the rate at which cells search for and locate the stimulatory spots, and arrest efficiency quantifies the ability to attach and arrest upon locating a spot. The encounter rate of memory cells is ∼1.5-fold higher than that of naive cells (Fig. 2E, 2F). This is in agreement with the ∼1.6-fold increased speed of memory cells on immobilized CCL21 (Supplemental Fig. 3C). The arrest efficiency of CD8^+^ hTm cells is 5.1-fold higher on 10-μm spots and 3.5-fold higher on 20-μm spots than that of naive CD8 T cells (Fig. 2G, 2H). Roughly 1 in 10 encounters leads to arrest on the spots in the case of naive CD8 T cells, whereas ∼40% of the encounters lead to arrest in the case of CD8^+^ hTm cells. As expected, multiplying these two parameters gives back the same on-rate of attachment as the value initially measured by a mathematically independent approach. Consistent with the observation on uniform stimulatory surfaces, there is no difference in SP of human CD4 naive and memory cells on stimulatory spots, with both having low values (Supplemental Fig. 4A–C). In contrast to humans, mouse CD8 naive and memory cells have the same SP (Supplemental Fig. 4D–F). Thus, CD8^+^ hTm cells have high SP both on uniformly coated surfaces and stimulatory spots.

### High SP of CD8^+^ hTm cells confers competitive advantage

**FIGURE 3.**
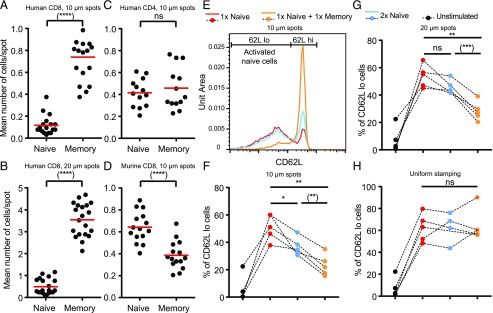
CD8^+^ hTm cells compete out naive T cells by preventing access to stimulatory spots because of high SP. (**A**–**D**) “1×” number (see *Materials and Methods*) of differentially labeled naive and memory cells were pooled and introduced into the channel with stimulatory spots. After 2 h, multiple fields along the channel were imaged for multiple donors. Mean number of cells attached per spot is plotted for each field: human CD8 T cells in (A) and (B), human CD4 T cells in (C), and murine CD8 T cells in (D). Mean value is shown in red. (**E**) Activation of human naive CD8 T cells assessed after 10–12 h of interaction with 10-μm stimulatory spots in a competitive setting by staining for CD62L. Having “2×” number of naive cells is a control case (blue) to compare against the scenario in which memory cells are also present (orange). Note that the histograms have the same number of naive cells represented. (**F**–**H**) Percentage of activated naive CD8 T cells, as measured by the percentage of cells with lower CD62L, under different competitive settings on 10-μm spots (F), 20-μm spots (G), and uniformly stamped surface (H). Lines that join the dots for each competitive setting represent the same donor. Data points for each competitive setting are color-coded [see top of (E) and (G)]. “1×” number of cells on 20-μm spots is considerably higher (see *Materials and Methods*). Channels with uniformly stamped surface received the same number of cells as the channels with 10-μm spots under the corresponding competitive settings. Each data point represents a separate donor and independent experiment in each plot. *p < 0.05, **p < 0.01, ***p < 0.001, ****p < 0.0001.

We reasoned that in a competitive setting with both cell types present in equal numbers, CD8^+^ hTm cells will prevent naive cells from arresting on spots. Expectedly, memory cells rapidly arrested on spots over the first 2 h, leaving the naive cells to explore areas outside the spots (Supplemental Video 3). The enrichment of CD8^+^ hTm cells, as per the relative number of cells on spots after 2 h, approximately matched their fold-increase in SP (i.e., on-rate of attachment, both on 10- and 20-μm spots) (Fig. 3A, 3B). Again, as predicted by lack of difference in on-rate of attachment (Supplemental Fig. 3A), there was equal sharing of spots between naive and memory human CD4 T cells (Fig. 3C). There was a marginal increase in on-rate of murine naive CD8 T cells (Supplemental Fig. 3D), which explains the slight enrichment of naive cells over memory cells on spots deposited with 2C11 (Fig. 3D).

We next investigated how SP of CD8^+^ hTm cells impacts activation of naive T cells in a competitive setting. We had shown that stimulatory spots can robustly prime human naive CD8 T cells to undergo full activation and multiple rounds of cell division (11). In this study, we looked at activation after 10–12 h under a competitive setting to begin with. A substantial fraction of naive cells shed CD62L because of continuous TCR signaling (Fig. 3E). These cells with lower levels of CD62L also show robust CD69 upregulation (11). With twice the initial number of naive cells, the fraction of activated cells expectedly reduces (Fig. 3E). However, with the presence of an equal number of memory CD8 T cells, the fraction of naive cells activated reduces even further (Fig. 3E). The same effect of suppression of naive cell activation by CD8^+^ hTm cells is observed across multiple donors and both on 10- and 20-μm spots (Fig. 3F, 3G). However, we did not see any suppression of naive cell activation in the presence of memory cells when immobilized anti-CD3 is available across the entire surface (Fig. 3H). This implies that memory cells do not secrete any suppressive soluble factor, nor do they participate in any contact-dependent mechanisms of suppression, either. The suppression of naive cell activation by memory cells can be accounted for by higher SP of memory cells, preventing access of naive cells to spots. However, the extent of suppression seen after 10–12 h is considerably less than what one would have anticipated based on the competitive advantage seen after 2 h (Fig. 3A versus Fig. 3F). We can attribute this to reduced durability (*t*_1/2_) of interaction of CD8^+^ hTm cells on the spots (11), allowing naive cells to gradually get access to the spots over longer periods of time. In fact, the extent of suppression, when compared against twice the number of naive cells, is in agreement with steady-state behavior predicted by the combined effect of on-rate (i.e., SP) and off-rate (i.e., inverse of durability) of interaction with stimulatory spots. Ultimately, the memory cells have ∼2-fold competitive advantage over naive CD8 T cells, resulting from ∼7-fold higher SP (Fig. 2) and ∼3.5-fold lower durability (11) on the 10-μm stimulatory spots. Overall, high SP of CD8^+^ hTm cells allows them to outcompete naive cells and prevent them from getting activated. Secondly, SP and durability combine to determine competition.

## Discussion

CD8^+^ hTm cells, uniquely, have higher SP compared with the naive counterparts in our experimental settings (Figs. 1, 2). This means CD8^+^ hTm cells more rapidly switch from low-adhesion scanning motility to high-adhesion immature IS once in contact with Ag. In a recent study, we also showed that CD8^+^ hTm cells predominantly formed mature IS, whereas naive cells predominantly formed IK that were durable because of confinement to the spot (11). SP, IS versus IK ratio, and durability are all distinct cell-intrinsic parameters of interaction with Ag.

How and why do the CD8^+^ hTm cells have high SP? We surmise a higher rate of productive TCR triggering events in CD8^+^ hTm cells, thus increasing the probability of calcium influx and immature IS formation (9, 15). Higher SP should naturally increase the fraction of precursors recruited to the response. It should also increase the synchrony in the response. Both of these attributes enhance the protective functionalities of memory cells. However, it is not clear why the property is unique to CD8^+^ hTm cells and absent in other memory cells examined. The competitive advantage of CD8^+^ hTm cells at the expense of naive counterparts (Fig. 3) provides an important clue to this conundrum. It is known that maintenance of the naive cell pool is important through the life of the host and is especially critical in aging (16). Reduction in naive cell number and repertoire, and especially that of naive CD8 T cells, is a hallmark of human aging (16). In mice, thymic output throughout life ensures maintenance of naive cells (17). In humans, thymic output is negligible in adults, and homeostatic proliferation maintains naive cells (17). We posit that CD8^+^ hTm cells compete out the naive cells to preserve the naive repertoire for when absolutely needed. In the case of human CD4 T cells, pre-existing cross-reactive memory cells are found at high abundance (18). The cross-reactivity in CD4 T cells likely arises at the molecular level, as longer antigenic peptides binding to class II MHC allows for variation outside of the few critical recognition and anchor residues (19). This obviates the need for high SP in human CD4 memory T cells. Cross-reactivity in CD8^+^ hTm cells manifests through high SP. Accordingly, original antigenic sin, a phenomenon wherein recruitment of naive cells against new variant epitopes is suppressed by memory cells against the original epitopes, occurs only in human CD8 T cells (20, 21) and not in mice (22). Whether higher SP solely drives this remains to be answered.

Finally, competition between naive and memory cells is not determined solely by SP and durability of interaction. Additional factors such as relative abundance, differential localization in the lymphoid organ, and preference for dendritic cell (DC) subsets likely influence the outcome in vivo. Memory cells may further impact DCs through cytokines, downregulation of surface Ag, and directly killing DCs upon engagement.

## Supplementary Material

Data Supplement
