# Supplementary material for: Cutting Edge: Synapse Propensity of Human Memory CD8 T Cells Confers Competitive Advantage over Naive Counterparts
Source: J Immunol. 2019 Jun 14;203(3):601–6. doi: 10.4049/jimmunol.1801687 (PMC6643047; doi:10.4049/jimmunol.1801687)
Supplement: Data Supplement [file JI_1801687.zip › JI_1801687_Supplemental_Figures_1.pdf]

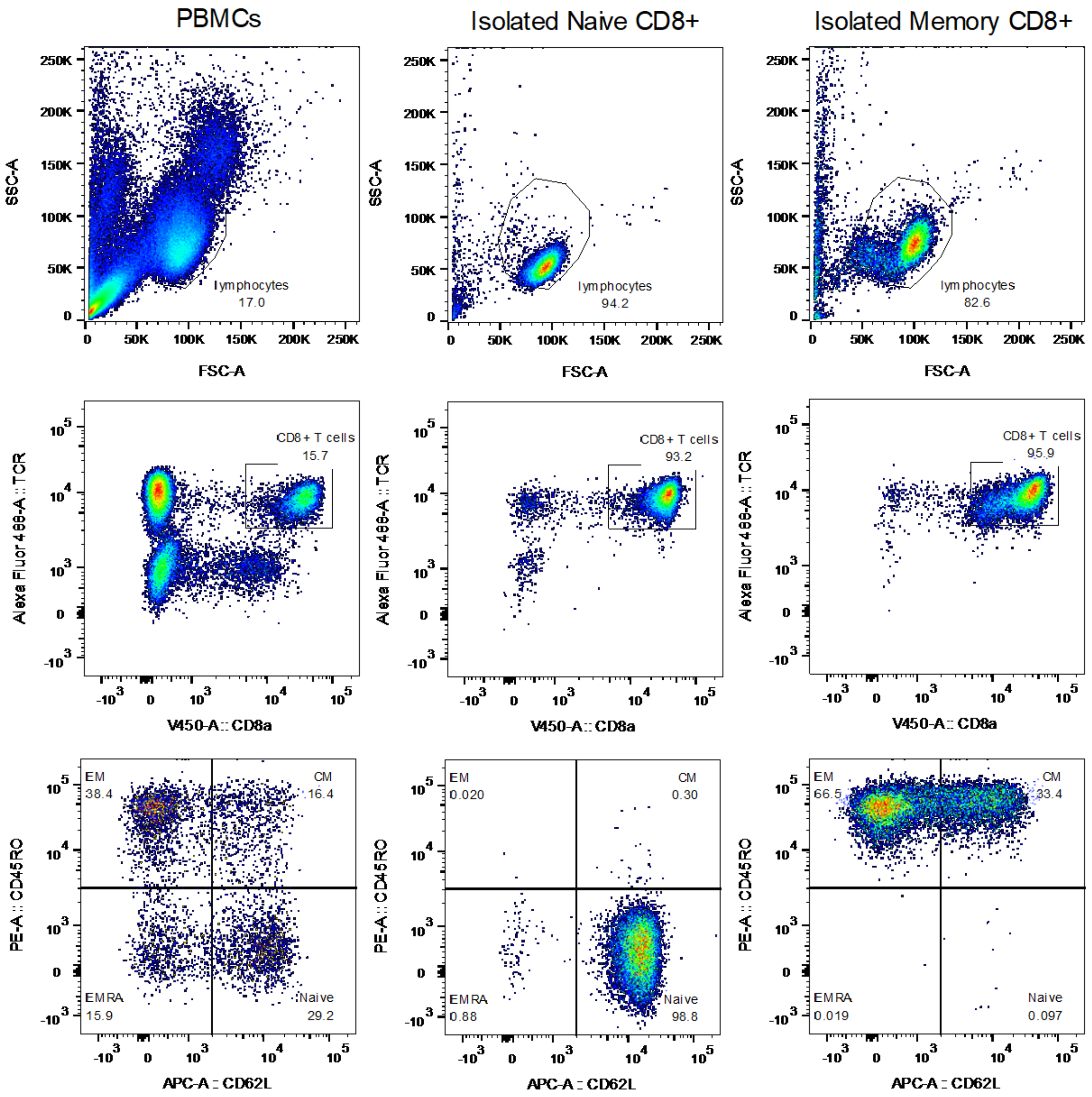

Supplemental Figure 1: Isolation of CD8$^+$ hTm and naïve cells from leukapheresis products. Total CD8 T cell population is isolated initially using the RosetteSep approach (from Stemcell Technologies). On the left column, we show the distribution of the subsets of interest in the initial PBMC pool through successive gates from top to bottom. The same gating strategy is used to show the enrichment of the desired subset (naïve in the middle and memory on the right) after the second step of isolation using the EasySep approach (from Stemcell Technologies). Overall, we get >90% purity of the desired subset. The primary contaminant appears to be CD4 T cells. Very few EMRA are present in the isolated naïve (typically <3%) and memory (typically <1%) cells. This is consistent with the absence of EMRA cells in human lymph nodes. Memory cell (EM and CM) contamination is <1% in the isolated naïve cells and vice versa. The isolated memory cells tend to have more dead cells (~ 10%) and some debris also accumulates in culture. The original relative distribution of central (CM) and effector (EM) memory cells is maintained after isolation. The relative distribution of CM and EM cells is comparable between peripheral blood and human lymph nodes. Essentially the same holds true for isolation of human CD4 naïve and memory subsets.

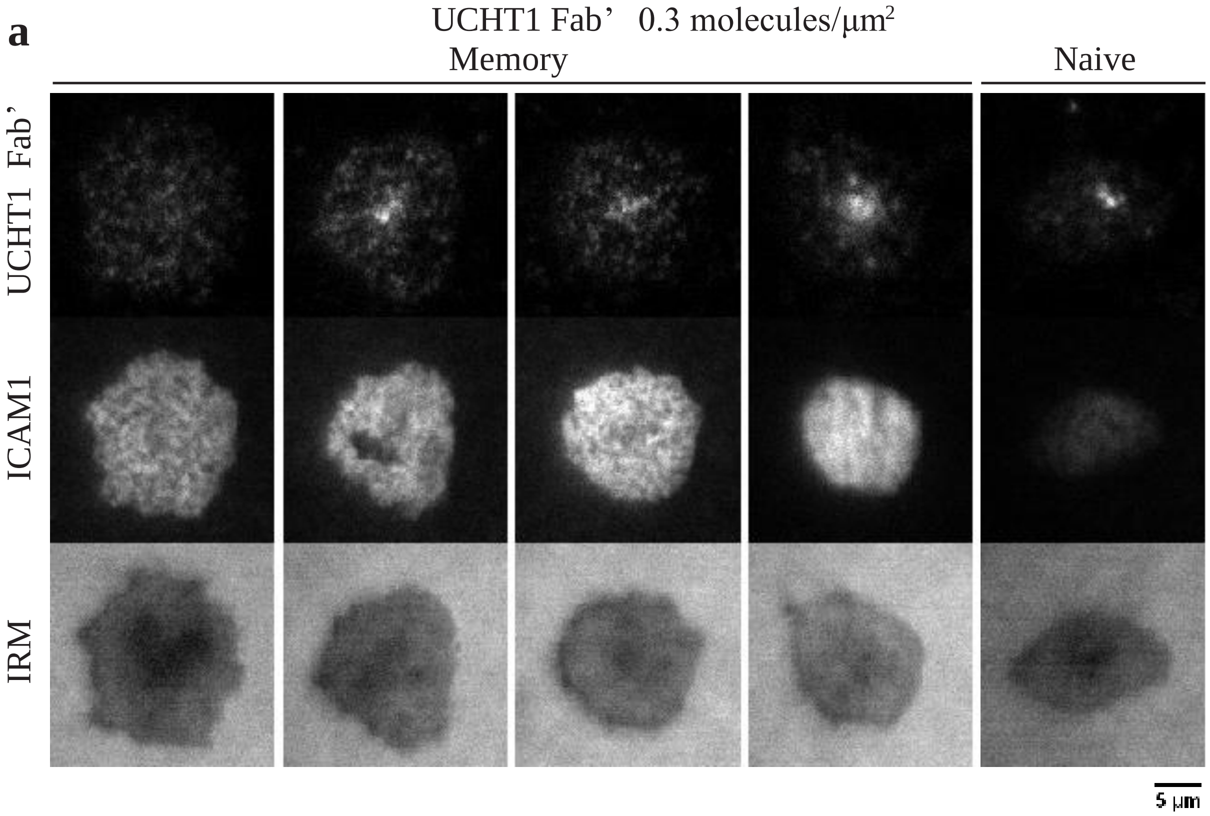

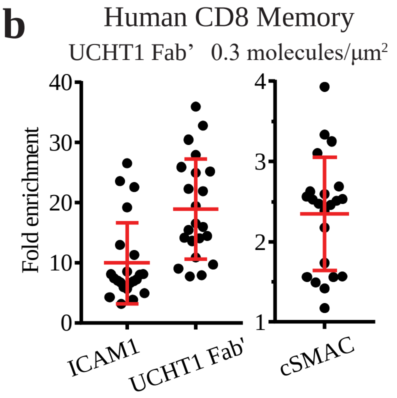

Supplemental Figure 2: Synapse formation of CD8⁺ hTm (4 columns on the left) and naïve (right most column) cells on very low density of UCHT1 Fab' (0.3 molecules/µm²) on SLBs. All cells with IRM footprint (bottom row) typical of synapses do show enrichment of ICAM1 (middle row) and UCHT1 Fab' (top row) at the synaptic interface. Most cells also show appreciable central accumulation of UCHT1 Fab' marking cSMAC. Except for the first cell (left most) in these examples, all cells show prominent cSMAC. However, the canonical pSMAC ring is absent as ICAM1 is not excluded at lower densities of UCHT1 Fab'. We found that 16 out of 22 CD8⁺ hTm cells that would have been scored as having formed IS or IK based on attachment footprint had a visually typical cSMAC even at 0.3 molecules/µm² of UCHT1 Fab'. Naïve cells accumulate less ICAM1 due to lower expression of LFA1. b) Enrichment of ligands due to continued accumulation in the interface of those cells with IRM footprint that is typical of IS or IK formation. Fold enrichment is the mean signal intensity in the interface relative to mean signal intensity in the surrounding region outside of the cell (i.e. background). For enrichment within cSMAC, mean intensity in the most intense region is expressed relative to that of the entire interface. Each data-point represents a cell. Mean and standard deviation are shown in red. All 22 cells had substantially enriched UCHT1 Fab' and ICAM1 in the interface compared to the surrounding. As ligand accumulation is the most prominent feature of IS or IK, we concluded that all memory cells selected in confocal micrographs had indeed transitioned through the immature synapse stage.

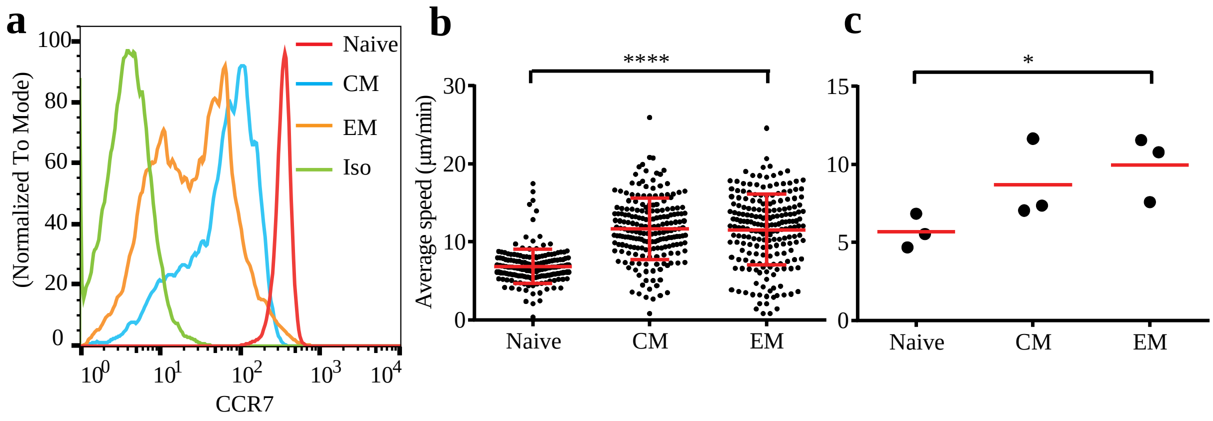

Supplemental Figure 3: a) Expression of CCR7 in naïve, central memory (CM) and effector memory (EM) human CD8 T cell subsets probed with CCR7-specific antibody (clone G043H7; Cat. No. 353205 from Biolegend). Representative of two independent experiments/donors. EM cells are thought of as being negative for expression of CCR7, the receptor for the chemokine CCL21 (Sallusto et al., Nature 1999). Comparison with isotype antibody and potentially improved reagent allowed for detection of expression in effector memory human CD8 T cells. b and c) Average speed of naïve, central memory (CM), and effector memory (EM) human CD8 T cells in response to immobilized CCL21 and ICAM1. When only ICAM1 is present <5% of the EM cells show motility, however along with CCL21 >80% of the cells show motility. Data points in b represent individual cell-tracks, whereas data points in c represent population means from separate donors. Note that central and effector memory cells move faster on immobilized CCL21 despite having reduced surface expression of CCR7.

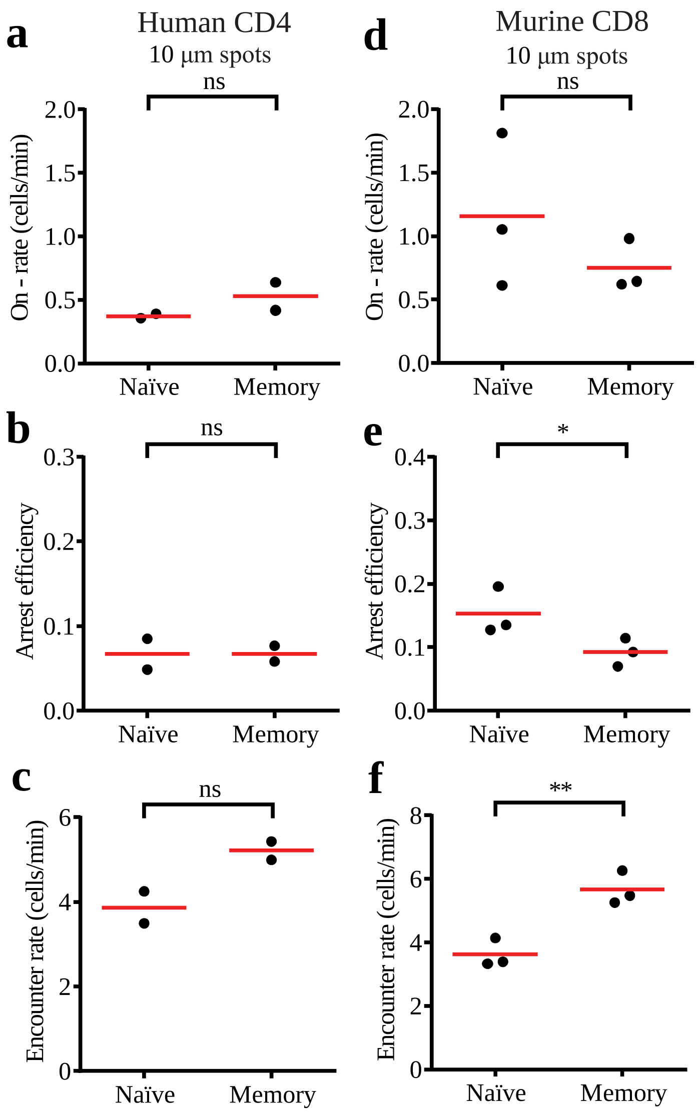

Supplemental Figure 4: Synapse propensity of human CD4 (a-c) and murine CD8 (d-f) T cells measured on 10 µm-wide stimulatory spots. Refer to Supplemental Methods section for details on the calculations for on-rate of arrest, arrest efficiency and encounter rate. There is no difference in any of the measured parameters in the case of human naïve and memory CD4 T cells. While arrest efficiency is higher for murine naïve CD8 T cells, it is offset by higher encounter rate of memory CD8 T cells, ultimately resulting in no significant increase on-rate of attachment and arrest. Each data point represents a separate donor and independent experiment in each plot. Mean value is shown in red. See Methods section for interpreting statistical significance.
