# Supplementary material for: Cutting Edge: Synapse Propensity of Human Memory CD8 T Cells Confers Competitive Advantage over Naive Counterparts
Source: J Immunol. 2019 Jun 14;203(3):601–6. doi: 10.4049/jimmunol.1801687 (PMC6643047; doi:10.4049/jimmunol.1801687)
Supplement: Data Supplement [file JI_1801687.zip › JI_1801687_Supplemental_Material_2.pdf]

Supplemental Video legends

Supplemental Video 1: Higher synapse propensity of $CD8^+$ hTm cells (green) compared to the naïve cells (red) on uniformly coated surface with CCL21, ICAM1 and low density of OKT3 (0.05 µg/ml used for coating). Equal numbers of differentially labelled naïve and memory cells were introduced into the Nunc well for imaging. The video also contains overlay of the IRM channel for visualize attachment footprints (dark gray). Time-ticker and scale bar are shown. Majority of memory cells attach and arrest (or decelerate) due to synapse formation. Majority of naïve cells only show chemokinesis in response to CCL21. Because of advection in the Nunc wells, most of these motile naïve cells drift from top end of the field to the bottom end.

Supplemental Video 2: Higher synapse propensity of $CD8^+$ hTm cells on 10 µm-wide stimulatory spots. Naïve cells are shown on the left and memory cells on the right. The video is an overlay of the spots (magenta) and cells imaged by DIC and IRM. The dark patches show attachment footprints, which happens when cells arrest on the spots. Time-ticker and scale bar are shown. Memory cells arrest on the spots at a faster rate, i.e. the number of spots with arrested memory cells increases rapidly. The field also contains appreciable number of non-responding and dead cells with Brownian motion among the memory cells.

Supplemental Video 3: Higher synapse propensity confers competitive advantage to $CD8^+$ hTm cells. Memory (green) cells rapidly arrest and attach on the 10 µm (left side) and 20 µm (right side) spots (in gray). This leaves very little area on the spots for naïve (red) cells to sample and arrest on to, as they have much lower synapse propensity.
